# Injectables in the Therapy of Mid-Portion Achilles Tendinopathy, a Descriptive Review

**DOI:** 10.3390/life15050824

**Published:** 2025-05-21

**Authors:** Daniela Poenaru, Claudia Gabriela Potcovaru, Miruna Ioana Sandulescu, Mariana Constantinovici, Delia Cinteza

**Affiliations:** Rehabilitation Department, Carol Davila University of Medicine and Pharmacy, 050474 Bucharest, Romania; claudia-gabriela.potcovaru@drd.umfcd.ro (C.G.P.); miruna.sandulescu@drd.umfcd.ro (M.I.S.); mariana.constantinovici@umfcd.ro (M.C.); delia.cinteza@umfcd.ro (D.C.)

**Keywords:** mid-portion Achilles tendinopathy, injection, intra tendon, peri tendon

## Abstract

Achilles mid-portion tendinopathy is defined as a painful thickening of the tendon, identified also on different imagistic examinations, occurring in sport people as well in inactively middle-aged individuals. The chronic and/or relapsing evolution interfere with daily living and alter the quality of life. Eccentric physical exercise is a cornerstone in her management and several injectable agents are used in clinical settings to reduce pain and improve function. According to the presumed pathogenic mechanisms, many classes of agents are in use: corticosteroids, protease inhibitors, sclerosing agents, pro-inflammatory agents, autologous products. The modalities of administration, either intra- or peritendon, the timing and number of sessions are displayed. Practical approach of chronic mid-portion Achilles tendinopathy consists of rest, tendon protection, eccentric exercise and therapeutical injections. The clinicians must choose between a spectrum of agents active on different pathogenic mechanisms, with benefits in the short and medium term. Future research may be focused on comparison between the different agents and on long term evolution.

## 1. Introduction

Chronic mid-portion Achilles tendinopathy is defined as a painful thickening of the tendon 2–6 cm proximally from the calcaneus insertion, often accompanied by paratendinopathy and featured as a structural abnormality on ultrasound and MRI. It usually spans over at least 3 months, producing pain, swelling and disability. It affects sportspeople as well as inactive middle-aged individuals.

Achilles tendinopathy arises with the occurrence of intrinsic and extrinsic factors. Intrinsic factors include, besides age and body structure and composition, aspects related to nutrition, metabolic disorders, diabetes mellitus or dyslipidemia. Extrinsic factors include abnormal loading, fatigue, improper loading, disuse, compression and exogen substances that may alter tendon structure (fluoroquinolones) [1].

At the cellular level, many mechanisms have been proposed to play a role in the inflammatory–degenerative cascade. Disturbance of metalloproteases and associated degenerative changes within the tendon, and ingrowth of new vessels and nerves from the ventral side of the tendon are some of the presumed mechanisms of pain [2]. Further, some inflammatory reaction was documented in non-ruptured Achilles tendons, consisting of immune-competent cells (macrophages, T lymphocytes, mast cells and natural killer cells) [3]. The role of inflammation in Achilles tendinopathy is re-evaluated as an important process in the persistence or recurrence of the disease [4,5]. Kager’s fat pad seems to play an important role in Achilles tendon metabolism, as biopsies of this structure revealed gene expression changes abutting to inflammation of Kager’s fat pad [6].

Management starts with a conservative approach and includes rest, local analgesia and physical agents (including extracorporeal shock wave therapy, laser therapy). Eccentric muscle training has become the dominant conservative intervention strategy over the past decade [7]. Patient education, tendon protection from loading activities and strengthening exercises are the first therapeutical steps [8]. Alfredsson, in 1998, organized eccentric training into a coherent program and since then, it has been considered a gold standard in conservative management [9]. In case of failure or recurrence, some other therapies are to be recommended, such as extracorporeal shock wave therapy, physical agents (ultrasound, laser, etc.) and local injections. Several innovative injectable therapies address pathogenic mechanisms and are of interest to the clinician. Although the evidence for injectable therapies is scarce, in clinical practice they are widely used and therefore, it is important to document their effectiveness [9].

The hypothesis of this paper is that injections are effective in the management of chronic or relapsing mid-portion Achilles tendinopathy. The objective of the paper is to document the main classes of injectable agents according to the pathogenic mechanisms and to follow their effectiveness in the short and long term.

The literature offers a few studies on a wide range of techniques that require systematization and comparison of effectiveness in the short and long term.

## 2. Materials and Methods

The primary objective of the actual research was to identify the spectrum of injectable agents for chronic and/or refractory mid-portion Achilles tendinopathy. Secondary objectives were to follow the methodology of therapeutic management of these agents within the general strategies, including number and timing of administrations, rest and exercise.

Our research followed the PICO criteria. The population to be studied was the pool of patients with chronic mid-portion Achilles tendinopathy (P), the intervention was any injectable approach (I), the comparison was with placebo (no therapy) or between injectable therapies, the outcomes were pain and functional improvement (primary) and adverse effects (secondary) [10].

We included research published until December 2024 on databases (Embase, Google Scholar, PubMed, Cochrane) following the MeSH terms: “midportion” AND “Achilles” AND (“tendinopathy” OR “tendonitis”) AND “injection”. All types of papers were included, i.e., pilot studies, case reports, longitudinal observational and controlled trials.

Inclusion criteria were papers in full text, available in English, on human subjects with mid-portion Achilles tendinopathy that received any form of injectable treatment, published in peer-reviewed journals selected according to the above-mentioned MeSH terms. Exclusion criteria were research on cell cultures and animal models, reviews, papers on insertional Achilles tendinopathy and papers on Achilles tendinopathy as a manifestation of a general, rheumatic disease.

Two independent reviewers (MIS and CGP) screened the literature and assessed each study to be included by reading titles, abstracts and full texts. They extracted the data in a table, and they compared the results. Any disagreement was solved through discussion with the team, until agreement was attained.

A total of 4215 papers were found and, after excluding duplicates, 3890 papers were left. We excluded reviews and were left with 1560 papers. Then, we excluded research on cell cultures and animal models, research on insertional tendinopathy, studies on Achilles tendinopathy as a manifestation of an inflammatory rheumatic or general disease (rheumatoid arthritis, spondilarthropathies, gout, etc.) and partial or total rupture of the tendon. There remained 50 papers to be screened in full text. We identified two papers referring to the same trial, so we mentioned them together, as a single source.

We decided to include all types of paper, assuming high heterogeneity, with the purpose of identifying the wide range of techniques used in management.

## 3. Results

**Figure 1:**
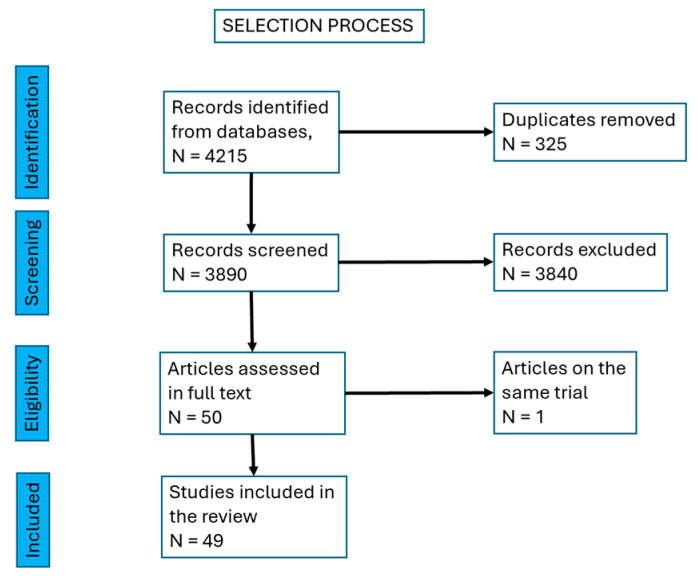
Selection process of the papers.

We finally selected 49 papers, with a total of 2222 Achilles mid-portion tendinopathies. There were 11 case series [11,12,13,14,15,16,17,18,19,20,21], 4 pilot trials [22,23,24], 6 retrospective interventional studies [25,26,27,28,29,30], 9 prospective, longitudinal, interventional, open-label, before-and-after trials [31,32,33,34,35,36,37,38,39], 16 prospective randomized controlled trials [40,41,42,43,44,45,46,47,48,49,50,51,52,53,54,55,56], one retrospective case report [57] and one non-randomized prospective trial [58] (Figure 1, Table 1).

We followed the pharmacological agents used in the management of tendinopathies (Table 2).

### 3.1. Corticosteroid Injections

Corticosteroid injections have the longest history of administration, based on their anti-inflammatory action. The presence of intra-tendon hypervascularization, visualized on Doppler images, supports the administration of corticosteroids [58]. There is an accepted consensus about the deleterious effects of intra-tendinous corticosteroid injections, as stated by a report on three cases of Achilles tendon rupture in athletics [59]. However, these agents are used in peritendinous administration, either fluoroscopically or ultrasound-guided. A retrospective study on 43 patients with a peritendinous injection of an amount of 1 mL corticosteroid and local anesthetic, with 2 years follow-up found no complications of the procedure. As for patient satisfaction, after 2 years, 40% reported significant improvement, 53% thought that their condition remained the same and 7% reported worsening. The authors favored this procedure because it allows injection into the pre-Achilles bursa [25]. Two prospective, randomized, double-blind, placebo-controlled trials with a total of 124 tendons with US-guided peritendinous injections (3 injections of 2 mL corticosteroid and anesthetic, at minimum 4 weeks interval) followed by eccentric training showed significant better results for corticosteroids in the short term (1, 2 and 6 months) and no severe adverse events at 2 years follow-up. Concerning the post-procedural recommendations, the eccentric program was started after the intervention, with refraining from strenuous physical activity for 3 months and gradual returning to sport activity. Adverse reactions in the peritendon administration appeared because of leaking within the surrounding tissues (skin depigmentation, skin atrophy), were mild and disappeared in time (three months). In the context of a complex rehabilitation program, corticosteroid injections are associated with physical exercise [40,56].

Neovascularization within the tendon is often present in chronic painful tendinopathy, although it was documented also in the pain-free tendons as a response to physical training in athletes. The “iceberg theory” underlines the fact that neovascularization is present long before clinical symptoms and ultrasound features [6,60,61]. It is a fact that neovascularization is present in 50–100% of patients with tendon pain at Doppler ultrasonography, compared with 0–30% in asymptomatic patients. The infiltration of nerve structures alongside this neovascularization has been suggested to play a role in the chronicity of pain from Achilles tendinopathy. Besides corticosteroids, another two procedures were proposed to attack the neovascularization: high-volume image-guided injection (HVIGI) and polidocanol.

### 3.2. High-Volume Image-Guided Injection (HVIGI)

The procedure implies injection with ultrasound guidance of a volume of 40–50 mL on the anterior aspect of the tendon, at the limit of Kager’s fat pad. The mechanism of action is believed to be mechanical stretching, breaking and occluding the neo-vessels and the accompanying nerves, leading to pain reduction and, accordingly, to functional improvement.

There are three modalities to use HVIGI: a combination of corticosteroid, local anesthetic and saline, a combination of anesthetic and saline and a combination of aprotinin, anesthetic and saline. Besides the mechanical effect of high volumes, the agents used may add their own therapeutical action, i.e., corticosteroids are anti-inflammatory agents and aprotinin is credited to act on the metalloprotease balance.

#### 3.2.1. Corticosteroid, Local Anesthetic, Saline

Three prospective, longitudinal, interventional, pre- and post-treatment studies included a total of 64 Achilles tendons that received an amount of 10 mL of corticosteroid, local anesthetic and saline followed by 40 mL saline on the anterior aspect of the tendon under ultrasound guidance. Post-intervention, relative rest for 3 days was followed by eccentric training. On the short and medium term, pain and function improved significantly, and ultrasound exam showed reductions in tendon thickness and neovascularization. On the long term, the achievements were stable, and the rate of failure was 6–7% [18,35,36]. However, a retrospective case series on 30 tendons with the same management reported a rate of success of 33% on pain and function at one year, possibly due to a peculiar selection of patients, with a longer duration of symptoms and multiple failures of conservative methods, as stated by the authors [21].

#### 3.2.2. Saline

The same administration technique was performed with a mixture of local anesthetic and saline, without corticosteroid. For the first 24 h, the patients were asked to refrain from strenuous activities and then, eccentric training was initiated.

A prospective, longitudinal, interventional case series on 14 tendons received 50 mL mixture (10 mL lignocaine and 40 mL saline) on ultrasound guidance and reported significant improvement in pain and function on the short and medium term, with a mean follow-up of 347 days. There was a failure rate of 14% of patients who went to surgery for persistent symptoms [20]. A prospective, randomized, double-blind trial on 80 tendons evaluated injection of 50 mL corticosteroid, local anesthetic and saline versus a 50 mL mixture of saline and local anesthetic on the anterior aspect of the tendon under ultrasound guidance. Pain and function scores improved in both groups at all moments (2–24 weeks), with no significant difference between them. Doppler was not detectable in 79% of patients in the high-volume group and in 30% of the placebo group and did not correlate with pain evolution. The authors concluded that a high volume without corticosteroid did not add any value [53].

#### 3.2.3. Aprotinin, Saline and Local Anesthetic

The mixture of aprotinin (a broad-spectrum protease inhibitor), saline and local anesthetic was proposed with the same technique, with a relative rest for the following 72 h and an eccentric program afterwards.

A case series of 94 athletic tendons that received 10 mL bupivacaine, saline and aprotinin (62,500 kIU) were monitored for pain, function and ultrasound imaging (grey-scale and Doppler). Patients were advised to refrain from heavy activity in the first 72 h and to start afterwards an eccentric exercise program. Two weeks later, if symptoms persisted, the procedure was repeated with hydrocortisone instead of aprotinin; in total, 60% of patients required it. After one year, 68% of patients returned to their previous level of activity (among them, 21% asked for a second injection) and 9% went to surgery (all of them after two injections) [19].

### 3.3. Sclerosing Agents (Polidocanol)

Polidocanol is a sclerosing agent used in the therapy of varicose veins (lower legs, esophagus, hemorrhoids) with few side effects. The active substance is an aliphatic non-ionized nitrogen-free surface anesthetic, with a molecular weight of approximately 600. It is available as a solution of 5 mg/mL. It acts primarily on the intimae layer in the vascular wall and has a local anesthetic effect. For Achilles tendinopathy, it was administered into the intra-tendinous vessels, as identified on ultrasound Doppler. The amounts of substance varied between 1 and 4 mL. During the procedure, the blood flow stopped and the pain disappeared but returned after a few hours. The patients were allowed free daily activities for one week and afterwards strenuous exercise was permitted [41].

Two early pilot studies from 2002 and 2003 followed a total of 21 tendons injected with 2 mL of polidocanol. The procedure could be repeated after 3–6 weeks, up to a total of four injections, until there were no more neovessels at Doppler exam. After a mean follow-up of 8 months, 16 out of 21 patients were reported to reduce pain and neovascularization [22]. A prospective randomized, controlled, double-blind trial on 20 tendons compared two intra-tendinous injections (3–6 weeks apart) of polidocanol versus lidocaine plus adrenaline. On the short term (3 months), there were significant better results in the polidocanol group for pain at loading activities and patient satisfaction, as well as for ultrasound examination (reduction of the hypervascularity areas). After the first injection of polidocanol, 50% of patients were satisfied, the other 50% were offered the second injection that produced complete resolution of the symptoms. Symptom evolution correlated with ultrasound hypervascularity [41].

For one single retrospective, longitudinal, interventional study (non-randomized, non-blinded) on 53 tendons that received up to five injections of polidocanol at 6-week intervals, according to symptoms resolution, the results were less optimistic. On the short term (6 weeks), 44% of patients experienced less pain, 42% the same amount of pain and 14% had more pain. On midterm follow-up (median 3.9 years) 53% of patients had additional therapies. Of those who did not seek any additional therapy, 32% experienced the same amount of pain. The controversial results of this study may be due to the retrospective design as it was not set up as a clinical trial. However, the authors conclude that the results are close to other therapies (corticosteroid injections and external triglyceryltrinitrate application) and outperformed by eccentric training. Finally, the conclusion of this report underlined the fact that a median of three sessions of ethoxysclerol failed to confirm the high beneficial value of sclerosing therapy [27].

### 3.4. Hyperosmolar Dextrose

Hyperosmolar dextrose is included in prolotherapy regimens, i.e., small volumes of an irritant agent are injected at multiple sites around the tendon and ligaments, with the aim to induce local inflammation, fibroblast proliferation, collagen synthesis and healing of the tissue. In the settings of Achilles tendinopathy, the amount of injected substance varied between 2 and 3 mL, containing a mixture of 25% dextrose and local anesthetic, under ultrasound guidance, into the lesion area, by 1 to 3 puncture sites. The procedure was followed by 2 weeks of heavy-loading activity restriction, and aspirin and other NSAIDs were banned, as they are disruptive for the inflammation and healing process.

In a prospective longitudinal interventional pilot trial on 36 tendons, the procedure was repeated every 6 weeks until the patient felt cured or there was no effect, up to a maximum of four injections. Six weeks after the last injection, there was significant pain reduction at rest, daily activity and strenuous exercise. Ultrasound imaging featured fewer anechoic clefts (by 43%), tendon texture improvements and reduced neovascularization (by 55%) [34]. In a prospective longitudinal interventional trial on 86 mid-portion tendinopathies, with 1 to 5 intra-tendinous injections, pain and structure on ultrasound exam improved significantly at 28 months [31].

### 3.5. Aprotinin

A mechanism proposed for the pathogenesis of tendinopathy is the change in the balance between different matrix metalloproteinases (MMPs) and their tissue inhibitor (TIMP). The balance between the two categories ensures the healing and its disruption (increase of MMPs and decrease of TIMP) leads to excessive collagen destruction and tendinopathy. Aprotinin is a natural serine proteinase inhibitor with a broad spectrum, which bonds reversibly with plasmin, kallikrein, trypsin and metalloproteinases. The first therapeutical indications were acute pancreatitis and hypofibrinolytic bleeding (1953); recently it was withdrawn. It was used for a long time as an off-label agent for intra-tendinous administration, based on collagenase inhibitory activity [62]. Two early case series (1993, 1997) with a total of 139 chronic Achilles mid-portion tendinopathies produced great rates of improvement (74–78%) and small percentage of failure (8–16%) [11,12]. The aprotinin regimens were four peritendinous palpatory-guided injections of molecular weight of either 20,000 kIU or 62,500 kIU.

Some years later (2005, 2006 and 2013), the interest for aprotinin raised again. A prospective, randomized, double-blind, placebo-controlled trial on 33 tendons compared 3 weekly intra-tendinous injections of either 3 mL (30,000 kIU) aprotinin and xylocaine with saline and xylocaine, both followed by eccentric training. Pain, function and patient satisfaction improved in both groups at 3 weeks, 1, 3 and 12 months, with a better but still not significant evolution for aprotinin group. The authors presumed that the lack of statistical significance may be due to the small size of the groups [42]. Another non-randomized trial on 128 tendons that received 5 weekly injections of 20,000 kIU aprotinin estimated a rate of 82% return to previous level of sport in 2–3 months [58].

Aprotinin was the subject of interest for a large team treating Achilles and patellar tendinopathies. In a retrospective cohort study on mid-portion Achilles tendinopathy, two-to-three peritendinous injections (palpatory-guided) produced 59% improvement and 3% failure in a patient satisfaction questionnaire [26]. The authors considered that allergies were the main adverse reactions and recommended 6 weeks delay between injections to reduce this risk [63].

It is of interest to mention that the interest for aprotinin faded in the last years, as no new attempts on this matter have appeared.

### 3.6. Hyaluronic Acid, HA

Viscosupplementation with HA for osteoarthritis and other degenerative joint diseases was included in guidelines. Furthermore, it offers therapeutic benefits in diseases of peri-articular structures, such as rotator cuffs and ankle sprains. Hyaluronic acid is credited to reduce the adhesions between the tendon and surrounding sheet or peritendon, to provide lubrication for tendon gliding and to promote healing.

Most of the literature mentioned the amount of 40 mg HA/2 mL with mannitol 0.5%. The administration was on the anterior side of the tendon under ultrasound surveillance. A prospective, interventional, longitudinal pilot on 17 Achilles tendons that received one HA injection reported improvement of pain, function and quality of life at 2 weeks and 12 weeks [24]. Three longitudinal, interventional, prospective trials included 70 Achilles tendons and reported pain, function and quality of life improvement at 1 to 12 weeks after 2 weekly peritendinous HA injections. Ultrasound exam revealed reduction in tendon thickness at 14 and 56 days [32,38].

Another prospective, open-label, multicenter clinical trial followed 25 Achilles tendons that received 3 weekly injections of a different HA (20 mg/2 mL; molecular weight 500–730 kDa) and reported improvement in pain and functions over a period of 90 days [39].

A prospective randomized controlled study on 59 tendons followed the results of two peritendinous weekly HA injections with 3 weekly sessions of extracorporeal shockwave therapy for pain and function at 4 weeks, and 3 and 6 months. Both groups improved significantly at all moments, with better results for the hyaluronic acid. A few mild adverse effects were noted in both groups, with rapid resolution [50].

### 3.7. Autologous Blood Injection

Researchers underlined the benefits of autologous blood injections in different tendinopathies, in a peritendinous administration. For the Achilles tendon, the procedure implied the extraction of an amount of 3 mL venous blood that was injected on ultrasound guidance on the anterior aspect of the tendon, preceded by 1 mL local anesthetic. Rest was advised for the next 48 h, with eccentric training afterwards. Two randomized controlled studies compared standard eccentric training alone with standard training with autologous blood injections. Two injections were delivered 4-to-6 weeks apart. One trial on 40 tendons found that at 6 weeks the improvement in pain and function were not statistically significant in the study group versus control and at 12 weeks the differences were of meaningful moderate size. The authors stated that the addition of autologous blood injections may be of value for standard eccentric training [46]. Another trial on 53 tendons reported no differences in pain and function between the two groups at 6 months [47].

Apart from pain of moderate intensity at the injection site, the procedure was reported to carry no adverse effects.

### 3.8. Platelet-Rich Plasma (PRP)

Platelets within PRP release various growth factors that play a role in tissue repair process. By far, the largest amount of research focused on PRP injections.

Four prospective, longitudinal, interventional, pre- and post-treatment trials included 112 tendons. All tendons received injections within the structural abnormalities visualized under sonographic exam. The amount of injected agent was between 3 and 6 mL. The regimen of administration varied between one session with one single skin puncture and one tendon penetration, one session with many skin punctures and multiple tendon penetrations, two sessions 3 weeks apart, everyone with one skin puncture and one tendon penetration, and three sessions 2 weeks apart, everyone with one skin puncture and multiple tendon penetrations. Post-procedural tendon protection with walker boot, heel lift or crutches for 24–48 h and gradual return to the physical exercise program during the next two weeks were advised. All trials reported significant pain and functional improvement in the short and medium terms, with maintenance at 2 and 4 years. MRI and ultrasound abnormalities showed improvement in 6 months [15,16,33,37].

Three retrospective, interventional studies on a total of 136 tendons reported controversial results. In a longitudinal case series, one PRP intra-tendon injection produced modest improvement (not significant) in pain and function and minor MRI evolution on an average of 13.9 months [14]. Failure to obtain symptom resolution after one injection warrant further administration. Two injections (30% of patients) or three intra-tendon injections (10% of patients) may be necessary to obtain 96% improvement [28]. Another retrospective, longitudinal, interventional study included 98 tendons that received one PRP injection intra- and peritendon under ultrasound guidance. At 6 months, 84% of patients were satisfied and 7% satisfied with reservations. In total, 8% were not satisfied and received a second injection (after 12 ± 6 months) and obtained significant improvement at final follow-up (41 ± 20 months) [29].

Three randomized, placebo-controlled, longitudinal trials (single- and double-blinded) on 299 tendons compared one PRP injection with saline (one skin puncture and many tendon penetrations) and found intra-group significant improvement in pain and function and no inter-group difference on short- and medium-term results (up to 6 months). One trial reported a huge drop-out rate in the long term (12 months) due to therapy failure and no difference between PRP and placebo in functional outcomes, except for tendon thickness that increased in the PRP group [43,44,48,55].

A randomized, controlled, longitudinal pilot trial on 20 tendons compared intra-tendon PRP injection with a standard eccentric program. Pain, function and quality of life were recorded at 6 weeks, and 3 and 6 months and revealed no significant differences between groups [23].

#### PRP with Tenotomy

Percutaneous needle tenotomy disrupts tendinopathic tissue, induces bleeding and promotes local healing. A prospective, longitudinal, observational case series on 34 upper and lower limb tendons (among them 12 Achilles tendons) associated percutaneous needle tenotomy with PRP intra-tendon injection and reported maximal functional and pain improvement during the first 4 months and maintenance of results for an average timeframe of 14 months. The authors postulated that PRP augmented the results of needle tenotomy [13].

### 3.9. Autologous-Conditioned Serum

Autologous-conditioned serum is a platelet- and growth factor-rich solution that stimulates angiogenesis and the healing process; it was first designed for osteoarthritis treatment [64].

A prospective, longitudinal, observational case series on 28 tendons that received one injection within the maximum pain point reported significant improvement in pain and function at 6 weeks. Two patients required a second injection (7%). After the procedure, 6 weeks of walker boot were advised, with complete rest in the first 2 weeks and afterwards an eccentric strengthening program [17].

A retrospective, comparative, non-randomized study on 50 tendons compared an eccentric training program with 3 weekly injections of autologous-conditioned serum. Both groups improved in pain and function on the short and medium terms, with significant better results for injection group. MRI at 6 months did not provide significant differences between groups [30].

### 3.10. Autologous Adipose-Derived Stromal Vascular Fraction (SVF)

Autologous adipose-derived stromal vascular fraction was credited with anti-inflammatory and immunomodulatory effects and used in tendinopathies management.

Two controlled, randomized trials compared a total of 100 tendons separated in two groups; they received either one PRP injection or one autologous adipose-derived SVF, administered intra-tendon and peritendon targeting the altered area. Pain, function and quality of life improved significantly in both groups, with a faster evolution for SVF group, observed at 15 days. At 6 months, MRI or ultrasound examination did not show differences between the two groups [49,52].

### 3.11. Comparing the Techniques

Several studies have focused on comparing some of the above-mentioned procedures.

A comparative, double-blinded, randomized trial on 60 tendons included three groups: HVIGI (one injection, a mixture of steroid, saline and local anesthetic), PRP (four injections every 2 weeks) and saline; all injections were performed on the anterior aspect, between the tendon and the peritendinous tissue just around the most affected area, under ultrasound guidance. After the procedure, the patients were allowed to walk and should refrain from sports and strenuous activities for 72 h. Afterwards, eccentric training was recommended. All groups improved at 6, 12 and 24 weeks in pain, function and patient satisfaction. Pain, function and satisfaction were improved more effectively in HVIGI than PRP at 6 and 12 weeks, and in PRP than in HVIGI at 24 weeks. Short-term (6 and 12 weeks) tendon thickness decreased more in HVIGI than in PRP, and at 24 weeks more in PRP. Overall, HVIGI seemed to act more rapidly and had the advantage of one single administration over PRP [51].

### 3.12. Other Therapies

In 2009, a single-blind, randomized trial compared results on pain, function and patient satisfaction of prolotherapy of the tender points around Achilles tendon with an eccentric training program. Prolotherapy consisted of tender points injection, usually located in the subcutaneous tissue around the Achilles tendon (as determined by clinical inspection) with a solution of glucose (20%) and local anesthetic. The number of injections varied between 4 and 12, according to pain resolution, on a weekly basis. The patients were distributed in three groups: prolotherapy alone, eccentric training alone and combined prolotherapy and eccentric training. All groups improved pain and function; inter-group comparison showed more rapid improvements (6 months) for the combined therapy, with equal results on long-term (12 months). The research offered also an economic point of view on health conditions and management. The economic analysis of the three therapies proved a substantial increase with the combined treatment, but when measuring the incremental cost-effectiveness ratio (ICER; i.e., additional costs divided by the additional benefit), the combined therapy offered the best value for the money [45].

## 4. Discussion

**Figure 2:**
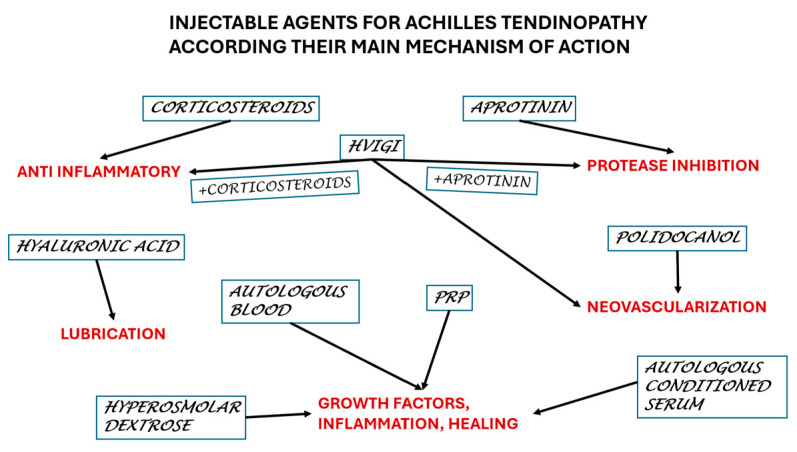
Selection of the agents according to the main mechanism of action.

We reviewed 49 papers on a total of 2222 Achilles mid-portion tendinopathies, which is a large pool with a diversity of therapeutical injections. This paper aims to offer the practitioners an overall picture of the actual knowledge to make a proper decision (Figure 2).

Although the number of studies reviewed is important, they deal with a diversity of techniques, making the amount of information for every technique rather scarce. In the last 15 years, PRP was studied in 13 trials, with a total of 771 patients, with variable results, from no effect to 91.6% satisfaction and 84.4% return to previous level of activities, with no adverse effects. Meantime, the lack of a control group may lead to confusing results. Other techniques add a small number of studies and patients, also with variable results, increasing the heterogeneity.

Another observation is that the number of randomized trials is low (13 randomized trials), the control group is saline and there are only two trials comparing different agents. In this situation, any comparison between products is disputable. On the other hand, 25 studies are longitudinal, before-and-after therapy, and carry the bias of superposing the natural healing tendency of the tendinopathy.

Corticosteroids carry the longest history of administration, based on the presumption that the leading phenomenon is inflammation. There is some evidence about repeated injections leading to tendon ruptures, either in intra-tendon or peritendon administration [65]. Clinical research supports clinical practice, as corticosteroid is recommended for rapid resolution of symptoms and rapid return to activity. In Achilles tendinopathy, peritendon administration of one to three injections, 4 weeks apart, offered good pain relief in the short term with no deleterious effects in the long term (2 years).

Corticosteroids were part of a special technique, high-volume image-guided injection HVIGI, that uses an amount of about 50 mL mixture (saline and local anesthetic) to be injected on the anterior aspect of the tendon, at the limit with Kager’s fat pad. Although the main mechanism is the disruption of peritendinous neovessels that invade the tendon substance, corticosteroids surely add an anti-inflammatory action, reducing neovascularization. This fact is evident when comparing HVIGI with corticosteroids and without corticosteroids, the first instance bringing more value to the patients. The technique is available for patellar and Achilles tendons, either insertional or mid-portion, especially when recalcitrant or chronic [66,67]. Another option for HVIGI is the addition of aprotinin, a collagenase inhibitor, possibly followed by a corticosteroid injection at 2 weeks interval.

Aprotinin in small volumes (3 mL) may be injected within the tendon or peritendon, targeting the affected area of the Achilles and patellar tendons, in three or four sessions, to obtain pain relief [68]. The risk of an allergic reaction imposed an interval of 6 weeks between the administrations. Aprotinin was found to be effective in peritendinous administration for rotator cuff tendinopathy [69].

Apart from corticosteroids, disruption of the neovessels may be obtained with polidocanol, a sclerosing agent injected peritendon, up to five injections at 6-week intervals. The ultrasound appearance of hyperemia guides the administration and the evaluation of the benefits. The technique offered good results for Achilles and patellar tendons [70]. For Achilles tendinopathy, it was reported to have a rate of success of around 44%, which might be sufficient to implement this therapy as a primary or secondary treatment option for some patients with symptomatic Achilles tendinopathy and intense hyperemia. However, two or three injections (6 weeks apart) are probably necessary to achieve comfort, and this might be unpleasant and time-consuming.

Hyaluronic acid is a largely used viscoelastic agent designed for intraarticular use and found to be effective for peritendinous administration, with a lubricant action and, possibly, a mechanical activity to break the peritendinous adhesions. It proved to be effective on peritendon administration, with three weekly injections. A narrative review underlined its effectiveness on pain reduction and functional assessment for peritendon injections in rotator cuff, elbow, hand, knee, ankle and foot tendinopathies [71].

Tendon healing could be triggered by local inflammation. Exogenous (dextrose) or endogenous, autologous agents may create an inflammatory reaction. Hyperosmolar dextrose, as a prolotherapy agent, is administered intra-tendon to produce irritation and, consequently, inflammation and healing. Dextrose prolotherapy is widely used with good results on pain and function in rotator cuff lesions and lateral elbow tendinopathy [72,73].

Autologous agents are derived from patient blood: autologous blood, autologous-conditioned serum and platelet-rich plasma. There are some opinions that the last two products are similar. These autologous agents release growth factors that promote healing. Autologous blood is usually injected peritendon, whereas autologous-conditioned serum and PRP are administered into the area of lesion. Autologous blood injections around patellar tendon were followed by statistically significant improvements in pain and knee function, whereas for lateral epicondylitis it proved to have no value [74,75]. Autologous-conditioned serum, a cell-free blood product, was used in supraspinatus tendinopathy with better results on pain and function compared to corticosteroids and in lateral epicondylitis in a pilot study with the early onset of pain-relieving action and long-lasting functional effects [76,77]. An important part of the literature deals with PRP injections, with most of the tendons being targeted for the therapy. Results are controversial, varying from valuable to no added value, which is comparative with non-PRP injections (for patellar tendinopathy) [78]. A recent meta-analysis stated that there is a trend towards pain reduction and functional amelioration from baseline with PRP injections for various tendinopathies (lateral epicondylitis, plantar fasciitis, rotator cuff tendinopathy, patellar tendinopathy, carpal tunnel syndrome) [79].

Currently, new therapies are explored, such as autologous adipose-derived stromal vascular fraction, which was initially designed for osteoarthritis. Further research is expected to deliver results.

The above-mentioned procedures were found to be safe, with a few transitory adverse reactions. The most fearful potential complication is tendon rupture; it may follow intra-tendon puncture, especially in corticosteroid administration.

It is important to define the relation between the injected therapies and the mechanical loading of the Achilles tendon. The intra-tendon injections were generally followed by a short period (up to 72 h) of tendon protection, either with crunches or walking boots. Afterwards, for a few weeks, usually 3–4 weeks, normal walking is permitted with refraining from heavy activities. Sport return is made gradually, according to symptomatology. Peritendon injections are followed by a few weeks of light walking and refraining from heavy activities; return to sport activity is gradual. In the absence of a good rehabilitation program, based on the golden standard of eccentric exercise and correct stretching, the risk of relapse and rupture increases.

Although considered second-line therapy, it should be associated with the first-line approaches. It is important to mention that injectable agents did not replace the first-line therapies, they complete them. All injectable therapies were part of a complex rehabilitation program that included necessary the eccentric training, starting quite rapidly after injection, after the first 24–72 h.

Ultrasound evaluation of the tendon added value to clinical and other imagistic examination (MRI) due to accessibility, reproducibility, costs and dynamic observation. Tendon appearance is important for diagnosing and evaluation, for therapeutic approach (needle guidance) and for follow-up.

## 5. Conclusions

Chronic and refractory mid-portion Achilles tendinopathy is subject to a multitude of injectable therapies as part of a complex approach. The first line of therapy is patient education, mechanical protection and triceps–Achilles complex strengthening. Failure or recurrence are subject to local injections, with a wide range of agents available. Results are discussed in short and medium terms; a few papers offer data on the long term (mostly 2 years). Corticosteroids used on the peritendon are important for short-term effects, with no deleterious effects in the long-term and are widely used. Intense hypervascularization may be subject to sclerosing agents. Regenerative techniques are trending and target the long-term evolution and remodeling of the tendon, to be documented in future research.

Despite the great number of published papers, guidelines avoid making strong recommendations for one or another therapy. In the rehabilitation guidelines, therapeutical exercise receives strong recommendations, while physical agents (as low-level laser therapy, iontophoresis, ESWT) receive recommendations based on inconsistent or limited-quality patient-oriented evidence. Recently, new evidence was added to support the use of injections alone or in combination with exercise therapy [80,81].

Future research with randomized trials to compare different agents is required and results in the long term should be monitored.

## Figures and Tables

**Table 1:** Studies on the injectable agents in mid-portion Achilles tendinopathy.

Author	Research Type	Modality of Administration	Agent, Dosing, Timing	Evaluation Tools	Timing	Results, Comments
Capasso, 1993 [11]	Prospective, longitudinal, interventional, case series, 77 tendons	Peritendinous	Aprotinin (62,500 kIU), 4 injections	Patient satisfaction	N/A	78% improvement (statistically significative) 7% failure
Aubin, 1997 [12]	Prospective, longitudinal, interventional, case series, 62 tendons	Peritendinous	Aprotinin (20,000 kIU), 4 injections	Patient satisfaction	N/A	74% improvement (statistically significative) 16% failure
Kleinman, 1983 [59]	Retrospective, case report, 3 tendons	Intratendinous	Corticosteroids	Pain Return to previous activities	2–3 weeks	Tendon rupture Avoid administration
Ohberg, 2002 [22]	Prospective, longitudinal, interventional, pilot, 11 tendons	Intratendinous, within the neovascularization area, possible repetition after 3–6 weeks, up to 4 injections	Polidocanol, 5 mg/mL, 2–4 mL	Ultrasound neovascularization Pain on VAS * Patient satisfaction	Baseline 6 months	8/10 patients reduced neovascularization and pain No side effects
Gill, 2004 [25]	Retrospective cohort study, therapeutic, 43 pts	Fluoroscopically guided corticosteroid injections into the space surrounding the Achilles tendon	Corticosteroid	Complications	2 years	Pain and function improvement statistically significant No major complications and one minor complication
Fredberg, 2004 [40]	Prospective, randomized, double-blind, placebo controlled, 24 tendons	Peritendinous, as close to the lesion as possible, US-guided	Corticosteroid, 3 injections (days 0, 7 and 21): 4 mL (20 mg triamcinolone + lidocaine)	Walking pain (NRS *), Pressure-pain detection thresholds, US * (tendon thickness)	Days 0, 7, 21, 28, 6 months	Tendon thickness and pain decreased in the CS group at 1 and 3 weeks and increased lightly at 6 months 4 days’ rest after injection. Complications: local atrophy, reversible.
Rochcongar, 2005 [58]	Non-randomized prospective trial, 128 tendons (athlets)	Peritendinous, 20,000 kIU, 5 weekly injections	Six groups: Aprotinine Physical exercise Orthosis NSAIDs per os Cryotherapy NSAIDs Surgery	Return to sport	2–3 months	82% of aprotinine patients returned to previous level of sport
Alfredson, 2005 [41]	Prospective, randomized, controlled, double-blind, 20 tendons	Intra-tendinous, right into the neovascularization area, ultrasound guided	Polidocanol (5 mg/mL) 1–4 mL versus blind (lidocaine + adrenaline), 2 treatments 3–6 weeks apart	Pain on activity, US * exam (neo-vascularization, tendon structure) Patient satisfaction	3 months	Significant improvement in all parameters in the polidocanol group No improvement in the blind group
Brown, 2006 [42]	Prospective, randomized, double-blind, placebo controlled, 26 pts, 33 tendons	Peritendinous, 3 weekly injections	Aprotinin + exercise versus placebo + exercise, 3 weekly injections	Primary outcome: VISA-A *, Secondary outcomes: Pain on VAS * Function (no. of single-heel raises) Return to previous level of sport Patient satisfaction	3 weeks, 1, 3 and 12 months	Both groups improved all parameters at all moments, with better results for aprotinin group, but not significant No side effects
Maxwell, 2007 [34]	Prospective, before and after, a sub-group of 23 mid-portion tendinopathy	Intra-tendinous, US-guided within the area of abnormality	2 mL of a mixture of 1 mL 2% lignocaine and 1 mL of 50% dextrose, giving a 25% dextrose solution One injection every 6 weeks, maximum 4 injections	GSUS *, color flow-DUS *, VAS * at rest, daily activity and strenuous activity	Baseline, 6 weeks after completion, 12 months	VAS at rest, daily and strenuous activity improved significantly after therapy US features improved
Chan, 2008 [35]	Prospective, longitudinal, interventional, pre- and post-treatment, 21 tendons	Peritendinous, ultrasound guidance, on the anterior aspect of the tendon	A mixture of 10 mL 0.5% bupivacaine hydrochloride and 25 mg of hydrocortisone acetate, followed by 4 × 10 mL of normal saline.	VISA-A * Pain on VAS *	Baseline 2 weeks 30 weeks	Significant improvement of pain and function on short and long term
Orchard, 2008 [26]	Retrospective, longitudinal, interventional, cohort study, 149 tendons	Peritendinous, palpatory guidance	A solution of 5 mL (aprotinin 30,000 kIU and 2 mL local anesthetic), 2–3 injections	Patient satisfaction questionnaire	3–54 months after the first injection	61% improved, 3% failure Adverse effects: itching (25%), rush (7%), uncommon (sweating, nausea, allergy, headache)
Yelland, 2009 [45]	Single-blind randomized clinical trial, 40 tendons	Tender points in the subcutaneous tissues adjacent to the tendon	Prolotherapy (5 mL solution glucose + local anesthetic) 4 to 12 weekly injections versus Eccentric training versus Eccentic training + prolotherapy	VISA-A * Patient satisfaction (Likert scale) Economic costs	Baseline 12 weeks (completion) 6 and 12 months	Intra-group: significant improvement at all moments Inter-group: at 6 months the combined therapy improved better Economic: combined therapy best value for money
Ryan, 2009 [31]	Prospective, longitudinal, interventional, 86 tendons	Intra-tendon, US-guided	Dextrose (2 mL 25% + lignocaine), 1–3 sites, repeated at 6 weeks if necessary	Pain Ultrasound aspect Number of injections	Baseline 6 weeks 28 months	Pain improved at all moments 28 months: US aspect improved Number of injections: between 1 and 5
De Vos, 2010 [43], De Jonge, 2011 [44]	Prospective, randomized, double-blind, longitudinal, placebo-controlled trial, 54 pts	Intra-tendinous	PRP (4 mL) versus saline, 3 different puncture locations.	VISA-A * Patient satisfaction, return to sports, Adherence to the eccentric exercises, Ultrasound exam	Baseline, 6, 12 and 24 weeks, One year	Both groups improved significantly at all moments No significant differences between groups No benefit for PRP group
Van Sterkenburg, 2010 [27]	Retrospective, longitudinal, interventional, 53 tendons	Intra-tendon, into the hypervascularity area	Polidocanol (2–4 mL), at 6 weeks interval and a maximum of 5 sessions	Pain on VAS *	6 weeks (short term), 3.9 years (midterm)	44% pain free 42% same amount of pain 14% worse pain No high beneficial value
Humphrey, 2010 [36]	Prospective, longitudinal, interventional, pre- and post-treatment, 11 tendons	Peritendinous, on the anterior aspect, ultrasound guided	A mixture of 10 mL 0.5% bupivacaine hydrochloride and 25 mg of hydrocortisone acetate, followed by 4 × 10 mL of normal saline	VISA-A * Ultrasound morphology	3 weeks	Significant improvement in all outcomes, with thickness reduction on US No serious event
Gaweda, 2010 [37]	Prospective, observational, before and after, 15 tendons	Intra-tendinous, US-guided within the hypoechogenic area	PRP, 3 mL, one injection	AOFAS * scale, VISA-A * scale US * assessment	Baseline, 6 weeks, 3 and 6 and 18 months	All parameters improved significantly at all moments
Finnoff, 2011 [13]	Prospective, longitudinal, observational, case series, 11 tendons	PRP, intra-tendon	US-guided percutaneous needle tenotomy and one PRP injection (2.5–3.5 mL)	Pain Function US * morphology	Baseline, 14 months (mean)	Significant improvement in all parameters. Maximum effect: 4 months PRP augmented the benefits of tenotomy
Owens, 2011 [14]	Retrospective, longitudinal, interventional, case series, 11 tendons	Intra-tendinous, US-guided,	PRP, 6 mL	FAAM *, FAAMS *, SF-8 *, MRI	Pre- and post-injection 13.9 (range, 10.1 to 19.5) months	Modest (not significant) improvement in clinical scales. Minor MRI changes.
Monto, 2012 [15]	Prospective, longitudinal, Interventional, case series, 30 tendons	Intra-tendinous, US-guided into the lesion, diamond injection pattern	PRP, 4 mL	AOFAS * MRI Patient satisfaction	1, 2, 3, 6, 12, and 24 months MRI at 6 months	Significant improvement in pain, function, return to activities MRI: structural improvement (statistically significant)
Pearson, 2012 [46]	Prospective randomized controlled trial, 33 tendons	Autologous blood injection, peritendinous	Eccentric training + autologous blood injection (peritendinous) versus eccentric training alone, second injection 6 weeks apart	Pain VISA-A * Ratings of perceived discomfort during and after the injection.	6, 12 weeks	6 weeks, not significant improvement in pain and function 12 weeks, moderate improvement Discomfort at injection site during the procedure and up to 48 h after
Ferrero, 2012 [16]	Prospective, longitudinal, interventional, case series, 30 tendons	Intra-tendinous, US-guidance	PRP, 6 mL, 2 injections 3 weeks apart	VISA-A * Pain (VAS *) US * Patient satisfaction	Baseline, 20 days, 6 months	Not significant improvement VISA-A, pain and US appearance at 20 days, significant improvement at 6 months, Moderate post-procedural pain (average 4 days)
Deans, 2012 [17]	Case series, prospective, 28 tendons	Intra-tendinous, into the maximum pain area	Autologous-conditioned plasma, one or two injections (6 weeks apart)	Pain Function Quality of life	Baseline, 6 weeks	Significant improvement in all parameters
Resteghini, 2012 [18]	Prospective, longitudinal, interventional, case series, 32 tendons	Peritendon, US-guided	40 mL (25 mg of hydrocortisone, 5 mL of 1% lignocaine and up to 40 mL of normal saline)	Pain (VAS *) VISA-A * Ultrasound exam	Baseline 1 and 3 months	Pain and function improved at all moments US exam improved at 3 months 6% rate of failure
Lynen, 2012 [32]	Longitudinal, interventional, prospective, single-arm, multicenter trial, 19 tendons	Peritendon, US-guided	Hyaluronic acid (40 mg/2 mL + mannitol), 2 weekly injections	Pain US * exam	Baseline 5 and 12 weeks	Significant improvement in pain and US structure None
Mautner, 2013 [28]	Retrospective, interventional, cross-sectional, 27 tendons	Intra-tendinous, US-guided	One or more injections, according to clinical evolution	Pain (at rest, function) Patient satisfaction	15 months (average)	All patients: not significant improvement. 96% mostly complete improvement
Maffulli, 2013 [19]	Prospective, longitudinal, interventional, case series, 94 tendons (athletes)	Peritendinous, on the anterior side, US-guided	10 mL mixture (bupivacaine and aprotinin 62,500 kIU), eventually followed by another injection, 2 weeks, with corticosteroid instead of aprotinin	VISA-A *, Ultrasound (GSUS *, DUS *) Return to athletic activity	Baseline, 2 weeks, 1 year	60% required a second injection One year: 68% success (return to previous level), 11% returned to a lower level 9% failure (surgery)
Bell, 2013 [47]	Prospective, randomized controlled trial, 53 tendons	Autologous blood injection, peritendinous,	Standard eccentric training alone versus standard eccentric training + autologous blood injections, 2 injections 4 weeks apart	VISA-A * Perceived rehabilitation (Likert scale) The ability to return to sport.	6 months	Both groups improved significantly, No differences between groups
Kearney, 2013 [23]	Randomized longitudinal, interventional, controlled trial, pilot trial, 20 tendons	Intra-tendinous administration (peppering, single-skin penetration and 5 penetrations of the tendon)	PRP (3–5 mL) versus excentric exercise program	VISA-A * Pain (VAS *) EQ-5D * for general health	Baseline, 6 weeks, 3 and 6 months	No significant differences
Filardo, 2014 [33]	Prospective, longitudinal, interventional, 37 tendons	Intra-tendinous, multiple perforations, US-guided	PRP, 5 mL, 3 injections every 2 weeks	Blazina score, VISA-A *, EQ-VAS * for general health, Tegner score	Baseline, 2 and 6 months, 4 years	Significant improvement on all parameters, in the short and medium term Maintenance at 4 years
Guelfi, 2014 [29]	Retrospective, interventional, 98 tendons	Intra- and peritendon injection, US-guided	PRP, one injection	Blazina score Pain VISA-A *	Baseline, 3 weeks, 3 and 6 months 50 months	91.6% satisfaction 8.4% failure
Wheeler, 2014 [20]	Prospective, longitudinal, interventional, pre- and post-treatment, case series, 14 tendons	Peritendon, on the anterior side, US-guided	HVIGI * 50 mL (10 mL lidocaine + 40 mL saline), one injection	Pain VISA-A *	Baseline 347 days mean follow-up	Significant improvement in pain and function 14% failure (went to surgery)
Krogh, 2016 [48]	Randomized placebo-controlled, single blind trial, 24 tendons	Intra-tendinous, ultrasound-guided injection.	PRP (6 mL) versus saline; peppering technique (3 to 4 skin portals and about 7 tendon perforations evenly distributed in the thickest part of the tendon)	VISA-A * Pain at rest, when walking, and when the Achilles tendon was squeezed. Ultrasound: color Doppler activity and tendon thickness	Baseline 3, 6 and 12 months	No differences between groups at 3 months for all parameters, except for the tendon thickness, which increased in PRP-group at 3 months A huge drop-out rate (54%) due to lack of results for both groups at 12 months
Girolamo, 2016 [49]	Prospective, controlled, randomized, 56 tendons	Intra-tendon and peritendon	Adipose tissue SVF versus PRP, one injection	Pain (VAS *) VISA-A * SF-36 * MRI/ultrasound	Baseline, 15, 30, 60, 120 and 180 days	Both groups improved at all moments SVF patients improved more rapidly (15 days) 6 months: imaging results were equal
Lynen, 2016 [50]	Prospective, randomized controlled, blinded-observer trial, 59 tendons	Peritendon	Hyaluronic acid HA (40 mg/2 mL + mannitol), 2 weekly injections versus ESWT (3 weekly sessions)	Pain (VAS *) VISA-A * Ultrasound exam	Baseline 4 weeks, 3 and 6 months	Intra-group: significant improvement in both groups Inter-group: HA group better result on all parameters Few adverse effects in both groups
Fogli, 2017 [38]	Prospective, open-label, single-center study, 34 tendons	Peritendon, on the anterior aspect	Hyaluronic acid (40 mg/2 mL + mannitol), 2 weekly injections	Pain US * exam Clinical symptoms Safety	Baseline Days 7, 14 and 56	Pain and clinical symptoms improved at all moments. On 14 and 56 days, reduction of tendon thickness No adverse effects
Boesen, 2017 [51]	Double-blinded, randomized prospective trial, 60 tendons	Peritendinous, on anterior aspect, ultrasound guided	HVIGI (one injection corticosteroid + anesthetic +saline) PRP (4 injections, every 2 weeks) Saline	Primary outcome: VISA-A * Secondary outcome: VAS *, satisfaction Ultrasound: tendon thickness and color Doppler	Baseline, 6, 12 and 24 weeks	Both HVIGI and PRP improve significantly parameters at all moments. HVIGI was more effective than PRP on pain, function and satisfaction at 6 and 12 weeks, but not at 24 weeks
Usuelli, 2018 [52]	Double-blind RCT, 44 pts	Intra-tendon and peritendon	One injection either of PRP or adipose tissue SVF	Pain on VAS * VISA-A * AOFAS * SF-36 * US * and MRI	15, 30, 60, 120 and 180 days	All patients improved significantly, SVF group with a faster evolution (15 days) 6 months: equally structural evolution
Boesen, 2019 [53]	Double-blind, randomized controlled, 28 tendons	Peritendinous, on the anterior side	HVIGI with and without corticosteroid (CS)	Primary outcome: VISA-A * Secondary: VAS * on weight-bearing, ultrasound (tendon thickness, Doppler), patient satisfaction	6, 12, 24 weeks	HVIGI with CS improved significantly in the short term and equally on medium term.
Von Wehren, 2019 [30]	Retrospective, longitudinal, comparative study, 50 tendons	Intra-tendinous, into the area of maximum pain	Autologous-conditioned serum (2 mL, 3 weekly injections) versus eccentric training	VISA-A * MRI (baseline and 6 months)	Baseline 6, 12 weeks, 6 months	VISA-A improved significantly better in autologous-conditioned serum group at all moments
Frizziero, 2019 [39]	Prospective, longitudinal, interventional trial, 26 tendons	Peritendinous, US-guided	Hyaluronic acid (20 mg/2 mL; 500–730 kDa), 3 weekly injections	Pain VISA-A * Quality of life (EQ-5D-5L *) US assessment	Baseline 14, 45 and 90 days	Significant improvement in pain and function up to 90 days Structural improvement at 90 days (statistically significative) No adverse effects
Der Vlist, 2020 [54]	Prospective, double-blind, randomized, placebo-controlled, 80 tendons	Peritendon, on the anterior side, into the area of maximum Doppler flow	50 mL versus 2 mL mixture of saline and 1% lidocaine	VISA-A * Patient satisfaction, return to sport, Doppler flow	2, 6, 12, 24 weeks	No benefit for the high-volume group No adverse effects
Ayyaswamy, 2020 [24]	Prospective, longitudinal, interventional, pilot study, 17 tendons	Peritendon, US-guided	Hyaluronic acid (40 mg/2 mL with 0.5% mannitol), one injection	Pain (VAS *) Manchester-Oxford Foot Questionnaire	Baseline 2 and 12 weeks	Significant improvement at all moments No adverse effects
Nielsen, 2020 [21]	Retrospective, longitudinal, interventional, case series, 30 tendons	Peritendon, on the anterior side, US-guided	One HVIGI (10 mL of marcaine, 0.5 mL of triamcinolonacetonid and 40 mL of saline)	VISA-A * US * exam	Baseline One year	33% of patients improved significantly
Kearney, 2021 [55]	Randomized placebo-controlled, longitudinal, multicenter clinical trial, 221 tendons	Intra-tendinous, a single skin portal and 5 penetrations of the tendon	PRP (3–5 mL) versus saline	VISA-A * Health-related quality of life assessed (EQ-5D-5L *)	Baseline 3 and 6 months	No difference between groups at 3 and 6 months
Johansson, 2022 [56]	Double-blinded randomized, controlled, placebo, 100 pts	Peritendinous, US-guided	Corticosteroid, 3 injections with an interval of at least 4 weeks versus placebo, followed by exercise	Primary: VISA-A * Secondary: pain on VAS *, global assessment, US exam (thickness, PDUS *)	1, 2, 3, 6, 12, and 24 months.	Both groups improved significantly. CS group improved better, 6 months. No deleterious effect of CS

NRS, numeric rating scale; VISA-A, Victorian Institute of Sports Assessment—Achilles; GSUS, grey-scale ultrasound; DUS, Doppler ultrasound; PDUS, power Doppler ultrasound; FAAM, Foot and Ankle Ability Measure; FAAMS, Foot and Ankle Ability Measure—Sports; SF-8, Short-Form health survey; EQ-5D, EuroQol 5-Dimension questionnaire; SVF, stromal vascular factor; AOFAS, American Orthopaedic Foot and Ankle Society; SF-8, measures health-related quality of life); HVIGI, high volume image guided injection, SF-36, 36-Item Short Form Survey; EQ-VAS, EuroQol-visual analogue scales; EQ-5D-5L, 5-level EQ-5D version. * means current item.

**Table 2:** Pharmacological agents used in the management of tendinopathies.

Agent	Main Mechanism	Administration
Corticosteroids	Anti-inflammatory	Intra-tendon—to be avoided Peritendon—advisable
HVIGI Corticosteroids Saline Aprotinin	Sclerosing agent for neovascularization +anti-inflammatory No other effect Proteolytic	Peritendon
Polidocanol	Sclerosing agent for neovascularization	Peritendon
Hyperosmolar dextrose	Inflammatory reaction to promote healing	Intra-tendon
Aprotinin	Proteolytic	Intra-tendon/Peritendon
Hyaluronic acid	Lubricating, breaking adhesions	Peritendon
Autologous blood	Inflammatory reaction to promote healing	Peritendon
PRP	Inflammatory reaction to promote healing	Intra-tendon
Autologous serum	Inflammatory reaction to promote healing	Intra-tendon
Autologous adipose-derived stromal vascular fraction	Inflammatory reaction to promote healing	Intra-tendon

## Data Availability

Data are available on the web pages of the databases.
